# Control and Elimination of Hepatitis C Virus Among People With HIV in Australia: Extended Follow-up of the CEASE Cohort (2014–2023)

**DOI:** 10.1093/ofid/ofae665

**Published:** 2024-12-17

**Authors:** Marianne Martinello, Joanne M Carson, Jeffrey J Post, Robert Finlayson, David Baker, Phillip Read, David Shaw, Mark Bloch, Joseph Doyle, Margaret Hellard, Ecaterina Filep, Samira Hosseini-Hooshyar, Gregory J Dore, Gail V Matthews

**Affiliations:** The Kirby Institute, University of New South Wales, Sydney, Australia; Prince of Wales Hospital, Sydney, Australia; The Kirby Institute, University of New South Wales, Sydney, Australia; Prince of Wales Hospital, Sydney, Australia; The Albion Centre, Sydney, Australia; School of Clinical Medicine, University of New South Wales, Sydney, Australia; Taylor Square Private Clinic, Sydney, Australia; East Sydney Doctors, Sydney, Australia; The Kirby Institute, University of New South Wales, Sydney, Australia; Kirketon Road Clinic, Sydney, Australia; Royal Adelaide Hospital, Adelaide, Australia; Holdsworth House Medical Practice, Sydney, Australia; Alfred Hospital, Melbourne, Australia; Burnet Institute, Melbourne, Australia; Alfred Hospital, Melbourne, Australia; Burnet Institute, Melbourne, Australia; The Kirby Institute, University of New South Wales, Sydney, Australia; The Kirby Institute, University of New South Wales, Sydney, Australia; The Kirby Institute, University of New South Wales, Sydney, Australia; St Vincent's Hospital, Sydney, Australia; The Kirby Institute, University of New South Wales, Sydney, Australia; St Vincent's Hospital, Sydney, Australia

**Keywords:** elimination, hepatitis C, HIV, men who have sex with men, treatment as prevention

## Abstract

**Background:**

Approximately 10% of people with HIV in Australia had active hepatitis C virus (HCV) infection prior to availability of government-subsidized direct-acting antiviral (DAA) therapy in 2016. This analysis evaluated progress toward HCV elimination among people with HIV in Australia between 2014 and 2023.

**Methods:**

The CEASE cohort study enrolled adults with HIV with past or current HCV infection (anti-HCV antibody positive) from 14 primary and tertiary clinics. Biobehavioral, clinical, and virologic data were collected at enrollment (2014–2016), follow-up 1 (2017–2018), and follow-up 2 (2021–2023). HCV treatment uptake, outcome, and HCV RNA prevalence (current infection) were evaluated. Death and HCV reinfection incidence and risk were assessed.

**Results:**

Of 402 participants, 341 (85%) had current HCV infection (RNA positive) at enrollment. Among the sample, 83% were gay and bisexual men, 13% had cirrhosis, and 80% had a history of injecting drug use (42%, past 6 months). DAA treatment was scaled up rapidly, with cumulative treatment uptake increasing from 12% in 2014 to 2015 to 92% in 2022 to 2023. HCV RNA prevalence declined from 85% (95% CI, 81%–88%) at enrollment (2014–2016) to 8% (95% CI, 6%–12%) at follow-up 1 (2017–2018) and 0.5% (95% CI, 0%–3%) at follow-up 2 (2020–2023). Sixteen reinfections occurred (incidence, 1.41 per 100 person-years; 95% CI, .81–2.29) as well as 30 deaths (incidence, 1.64 per 100 person-years; 95% CI, 1.11–2.34). HCV reinfection incidence declined over time while mortality remained stable.

**Conclusions:**

Universal access and rapid DAA uptake were associated with a dramatic reduction in HCV prevalence and reinfection incidence among people with HIV to levels consistent with microelimination.

**Registration:** NCT02102451 (ClinicalTrials.gov).

## Teaser

Universal treatment access and rapid direct-acting antiviral uptake were associated with a dramatic reduction in hepatitis C virus prevalence and reinfection incidence among people with HIV in Australia to levels consistent with microelimination.

In 2016, the World Health Organization called for the “elimination of viral hepatitis as a public health threat” by 2030, with the development of direct-acting antiviral (DAA) therapy generating impetus for hepatitis C virus (HCV) elimination [1]. Elimination targets included 80% treatment uptake among those eligible, 90% reduction in new infection incidence, and 65% reduction in liver-related mortality [2]. In 2022, World Health Organization guidance for HCV elimination validation outlined new outcome targets focused on current population-level disease burden, including absolute population-based incidence measures (rather than relative declines from 2015) for HCV-related mortality (<2 per 100 000 total population per annum) and HCV incidence (<5 per 100 000 total population per annum and <2 per 100 person-years [PY] among people who inject drugs) [2]. People with HIV were identified as a key population for HCV elimination, given the burden of infection and associated morbidity and mortality due to accelerated liver disease progression. In 2015, an estimated 2.3 million people with HIV were coinfected with HCV, with end-stage liver disease a major cause of death [3–5].

In Australia, approximately 26 000 people had HIV in 2016, and 10% were estimated to have HCV infection [6, 7]. On 1 March 2016, government-subsidized DAA therapy was made available to all adults with chronic HCV infection, regardless of liver disease stage or drug and alcohol use, via the Pharmaceutical Benefits Scheme, with authorized prescribing by general practitioners and specialists [8]. The Control and Elimination of HCV From HIV-Infected Individuals Within Australia (CEASE) cohort was established in 2014 through a network of high-caseload health services across Australia to evaluate the impact of DAA therapy on the burden of HCV infection among people with HIV. We hypothesized that HCV elimination would be feasible in this population, given the relative population size [7], high proportion diagnosed and linked to care [6], availability of harm reduction for people who inject drugs [9], and broad DAA access [8].

The aim of this analysis was to evaluate progress toward HCV elimination among people with HIV in Australia between 2014 and 2023 following universal access to DAA therapy.

## METHODS

### Study Design and Setting

CEASE was a prospective cohort study evaluating the impact of DAA scale-up on HCV burden among people with HIV in Australia. The study design has been described previously [10]. In brief, adults (age ≥18 years) with HIV infection who were anti-HCV antibody positive were eligible for enrollment, irrespective of HCV RNA status. Participants were enrolled from 14 high-caseload sites in primary care (n = 12) and tertiary viral hepatitis clinics (n = 2) in New South Wales, Queensland, South Australia, and Victoria (Supplementary Table 1). Enrollment commenced on 31 July 2014, with last follow-up on 15 February 2023.

### Study Assessments

Study visits were undertaken at enrollment (31 July 2014–22 March 2017; only 5 participants enrolled in 2017), follow-up 1 (26 May 2017–31 May 2018), and follow-up 2 (13 April 2021–15 February 2023). At each visit, participants completed a biobehavioral questionnaire, underwent transient elastography (FibroScan), and had a dried blood spot sample collected (for central HCV RNA testing; see Supplementary material). The questionnaire included demographics, drug and alcohol use, and sexual behavior (gay and bisexual men only), as well as HCV acquisition, knowledge, and treatment willingness (enrollment only). HCV treatment (pre- and postenrollment), such as start and stop dates, regimen, outcome, and relevant local laboratory results (eg, HCV RNA, HIV RNA, CD4), were collected and updated in real time. Anti-HCV antibody screening and confirmatory HCV RNA testing are provided as standard of care for all people with HIV in Australia. Participants with current HCV infection (detectable HCV RNA) were offered treatment at any time per standard of care, including by clinicians providing HIV care in primary and tertiary settings. Follow-up CEASE visits were undertaken, regardless of HCV RNA status at enrollment or treatment initiation during the study period.

### Study Oversight

All participants provided written informed consent before study procedures and received $30 renumeration for each study visit. The protocol was approved by St Vincent's Hospital, Sydney Human Research Ethics Committee (primary study committee), and was conducted according to guidelines of the Declaration of Helsinki and the International Conference on Harmonization Good Clinical Practice, as well as local regulatory requirements. The study was registered with ClinicalTrials.gov (NCT02102451).

### Statistical Analysis

The proportion with current HCV infection (detectable HCV RNA) was calculated throughout the study period by individual HCV RNA test results (local and central), date of HCV diagnosis, and HCV treatment outcome. Participants who died or revoked consent were censored at date of death or last follow-up.

Reinfection incidence was assessed among participants with a negative HCV RNA test result or documented sustained virologic response (SVR) and at least 1 subsequent HCV RNA test and was calculated with person-time of observation (per 100 PY). Time at risk commenced at enrollment (HCV RNA negative) or end of treatment (achieved SVR) and was censored at date of reinfection (estimated), death, or last follow-up. Estimated date of reinfection was calculated as the midpoint between the date of last negative HCV RNA (or end of treatment) and first positive HCV RNA. Reinfection was defined by the presence of quantifiable HCV RNA after SVR or the presence of quantifiable HCV RNA between end of treatment and posttreatment week 12 with detection of an HCV genotype/subtype that was distinct from the primary infecting strain, with confirmation of heterologous virus on sequencing if available. Attributable risk of injecting drug use during follow-up on reinfection incidence was calculated.

Mortality incidence was assessed among participants with at least 2 study data points and calculated with person-time of observation (per 100 PY). Time at risk commenced at enrollment and was censored at date of death or last follow-up. Confidence intervals with Poisson distribution were calculated. Causes of death were categorized by HIV Cohorts Data Exchange Protocol codes (Supplementary material). Parametric survival models were employed to assess factors associated with death and reinfection [11]. Selection of the underlying distributions for the hazard function was guided by the Akaike information criterion; an exponential distribution was applied to model factors associated with death; and a Gompertz distribution was applied to model factors associated with reinfection. Unadjusted and adjusted hazard ratios (aHRs) with corresponding 95% CIs were estimated. Nonparametic models were used to visualize cumulative hazard for reinfection (Nelson-Aalen estimator) and survival probability (Kaplan-Meier estimator). Analysis was performed with R version 4.3.1 (analysis: epiR, flexsurv, survminer; data visualization: ggplot2, ggalluvial) [12].

## RESULTS

### Population Characteristics at Enrollment

In total, 402 participants with HIV and evidence of current or past HCV infection (anti-HCV antibody positive) were enrolled, of whom 95% were male, 83% identified as gay or bisexual men, and the median age was 49 years (IQR, 43–55; Table 1). The majority were receiving antiretroviral therapy (n = 377, 94%), with a median CD4 count of 596 × 10^6^/L (IQR, 440–810). Most (80%) reported a lifetime history of injecting drug use (median age at first injecting, 27 years; IQR, 20–35), and 37% had injected drugs in the past 6 months (predominantly stimulants, 32%). Among 335 gay and bisexual men, 65% reported sex with 1 or more male partners (regular or casual) in the past 6 months; 52%, condomless anal intercourse with casual male partners; and 32%, group sex in the past 6 months.

**Table 1.** Participant Characteristics at Enrollment (N = 402)

Demographic/Clinical Characteristic	Median (IQR) or No. (%)
Age, y	49 (43–55)
Gender	
Male	382 (95)
Female	15 (4)
Transgender	4 (1)
Not specified	1 (0)
Sexual identity	
Heterosexual	60 (15)
Bisexual	28 (7)
Gay or lesbian	310 (77)
Prefer not to answer	4 (1)
Gay and bisexual men	335 (83)
Country of birth	
Australia	258 (64)
Europe or United Kingdom	42 (11)
New Zealand or Pacific Islands	30 (6)
Other or unknown	72 (18)
Ethnicity	
White	344 (86)
Asian	26 (6)
Hispanic	8 (2)
Other or not specified	21 (5)
Income source	
Full- or part-time employment	164 (41)
Government support payment ^a^	205 (51)
Retirement fund	13 (3)
Other or unknown	20 (5)
Completed higher education ^b^	207 (52)
Lives alone	176 (44)
History of anxiety and/or depression	176 (44)
Injecting drug use	
Ever	321 (80)
Within the last 6 mo	148 (37)
CD4 count, 10^6^/L	596 (440–810)
Combination antiretroviral therapy	377 (100)
HCV RNA detected	341 (85)
Mode of HCV acquisition: clinician assigned	
Injecting drug use	217 (54)
Male-to-male sex	112 (28)
Heterosexual contact	15 (4)
Other or unknown	67 (16)
Cirrhosis ^c^	51 (13)
Liver stiffness measurement, kPa	6.3 (5.0–8.4)
Hepatitis B	
Surface antigen positive	19 (5)
Core antibody positive	117 (29)
Surface antibody positive	212 (53)

Abbreviation: HCV, hepatitis C virus.

^a^Government support payments include pensions, disability, unemployment, and study support payments.

^b^Completed higher technical, college, or university degree.

^c^Of liver cirrhosis cases, 57 of 87 were diagnosed by transient elastography and 1 of 58 by liver biopsy.

### Impact of DAA Scale-up on HCV Infection

**Figure 1.**
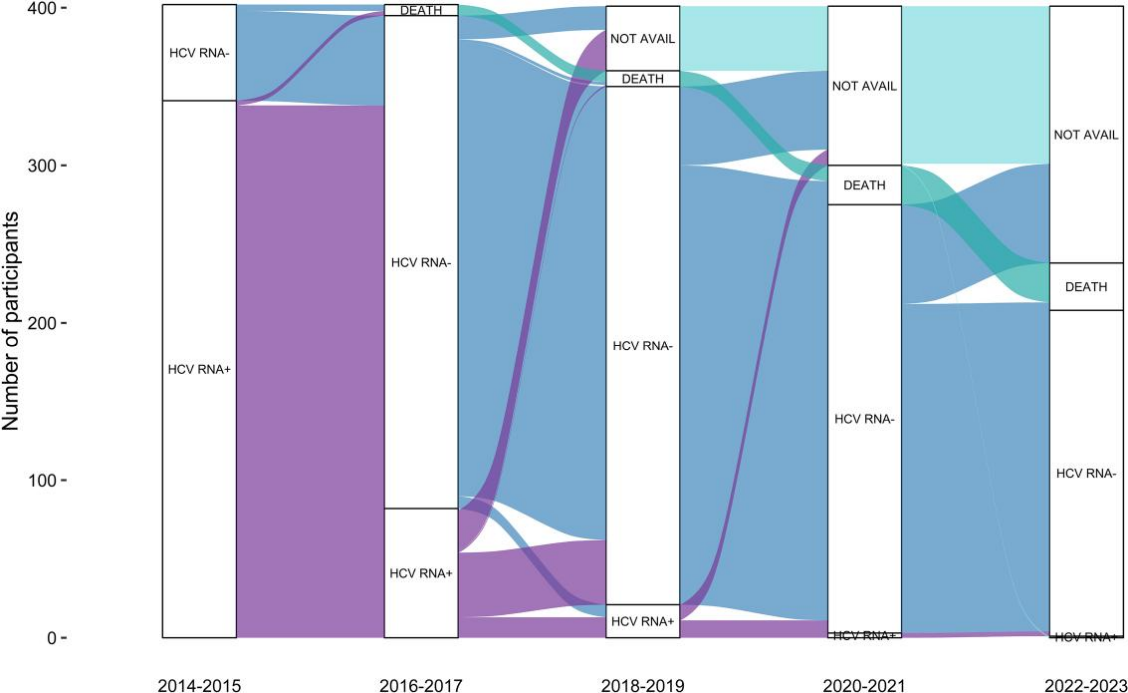
Alluvial plot of HCV status. The plot visualizes transitions between HCV status and states over the study period. Positive (+) and negative (–) signs denote whether HCV RNA was detected or not. “Not avail” indicates that no HCV RNA test was available. HCV, hepatitis C virus.

**Figure 2.**
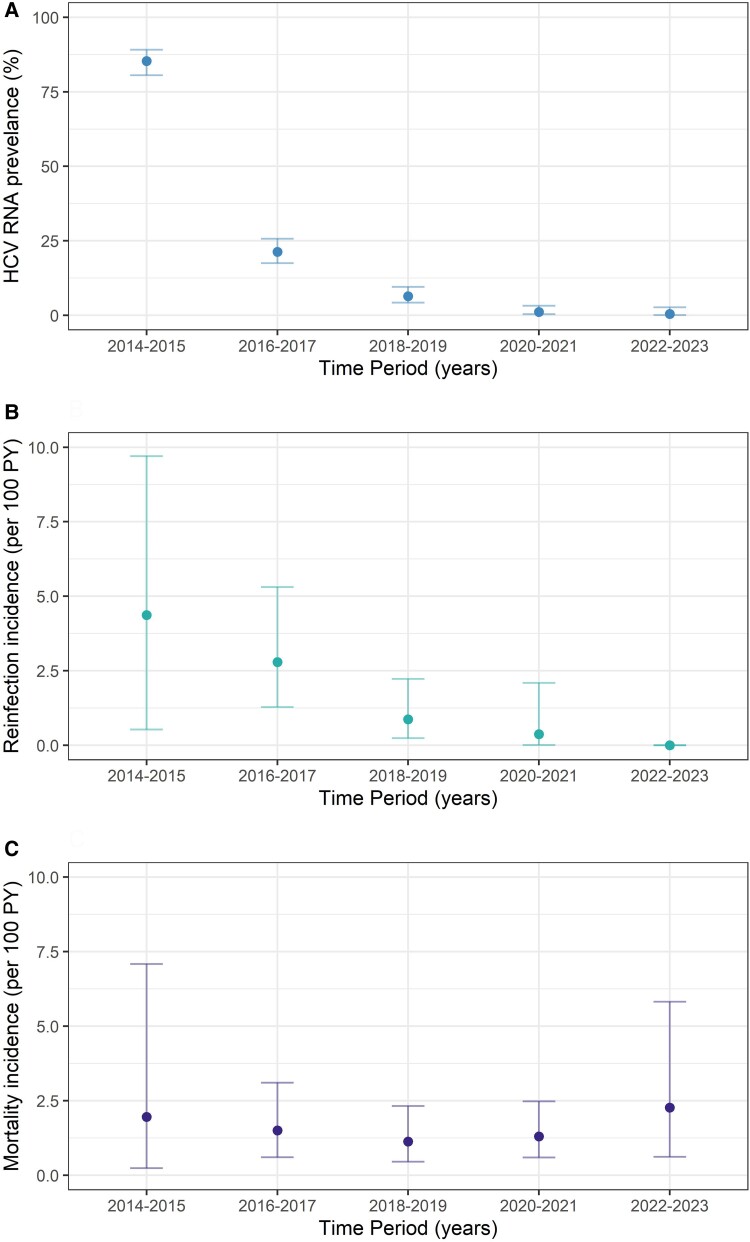
Burden of HCV infection, reinfection, and death among people with HIV in the elimination era (2014–2023). *A*, HCV RNA prevalence. *B*, Reinfection incidence. *C*, Mortality incidence. Error bars indicate 95% CI. Abbreviations: HCV, hepatitis C virus; PY, person-years.

At enrollment, 341 (85%) participants had current HCV infection (Figures 1 and 2*A*). DAA treatment was scaled up rapidly from 2016, with most (71%) treated in the first 9 months of government-subsidized access (Supplementary Figure 1). Among those with current HCV infection, cumulative treatment uptake increased from 12% in 2014 to 2015 to 91% in 2017 to 2018 and 92% in 2022 to 2023. Treatment adherence was high, with 5% reporting noncompletion (range, 7–28 missed doses). The proportion with current HCV infection declined from 85% (95% CI, 81%–88%) at enrollment (2014–2016) to 8% (95% CI, 6%–12%) at follow-up 1 (2017–2018) and 0.5% (95% CI, 0%–3%) at follow-up 2 (2022–2023; Figures 1 and 2*A*).

Only 2 participants had current HCV infection at follow-up 2: 1 had HCV reinfection (second documented reinfection) and 1 had primary infection, which was successfully treated by study end. Among 312 participants who received treatment, 9% were retreated (including 6 participants who received 2 courses of retreatment and 1 participant who received 3 courses).

Importantly, high cumulative treatment uptake (96%) with corresponding low HCV RNA prevalence at last review (4%) was seen among people lost to follow-up after 2018 (after initial DAA scale-up; Supplementary Table 2); characteristics of those who completed CEASE follow-up visits are shown in Supplementary Table 3. Characteristics of those who were and were not retained in follow-up were similar, with no difference in current HCV infection, receipt of HCV treatment, or risk behavior (Supplementary Table 4).

### HCV Reinfection and Transmission Risk Behavior Among People With HIV

**Figure 3.**
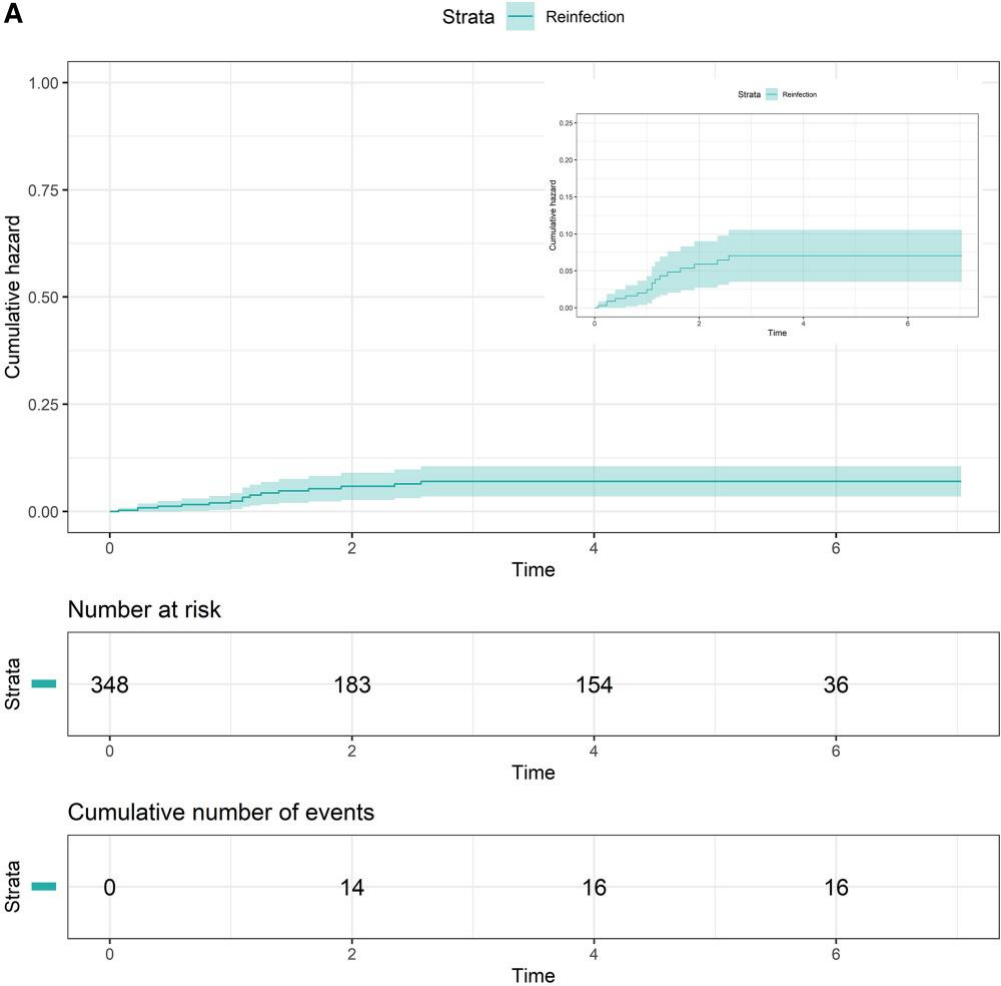

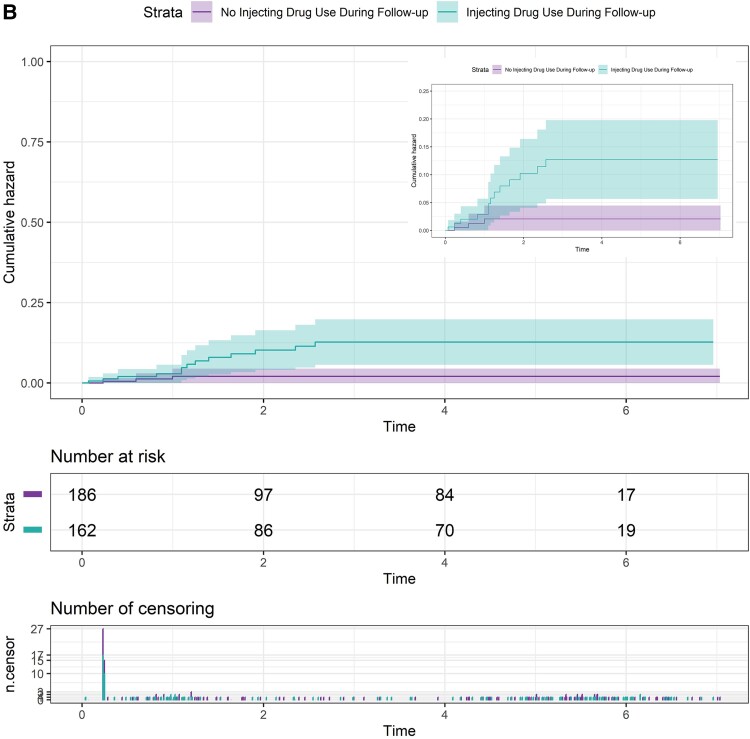
Cumulative hazard of hepatitis C virus reinfection. *A*, Overall. *B*, By injecting drug use status during follow-up. Shading indicates 95% CI.

Reinfection incidence was 1.42 per 100 PY (95% CI, .81–2.31), which declined over time (16 reinfections among 348 participants; total follow-up time for reinfection, 1123.8 PY; median follow-up time per person, 3.29 years [IQR, 0.90–5.59]; Figures 2*B* and  3*A*). Among individuals with reinfection, the median age was 46 years (IQR, 39–54), 88% (14/16) identified as gay or bisexual men, and 81% (13/16) reported injecting drug use during follow-up. Median time to reinfection was 9 months (IQR, 3–14).

Injecting and noninjecting drug use remained stable over the study period, while sexual behavior among gay and bisexual men changed (Supplementary Figure 2). The proportion reporting injecting drug use in the past 6 months was 39% at follow-up 1 and 29% at follow-up 2 (for trend, *P* = .119). The proportion identifying injecting drug use in the past month was 26% at enrollment, 29% at follow-up 1, and 24% at follow-up 2 (for trend, *P* = .459). Among those who reported injecting in the last month, stimulants were the predominant drug used (enrollment, 19%; follow-up 1, 24%; follow-up 2, 20%). While the proportion indicating condomless anal intercourse with casual male partners in the past 6 months declined (56% at enrollment, 47% at follow-up 1, and 46% at follow-up 2; for trend, *P* = .004), the proportion reporting group sex in the past 6 months remained stable (32% at enrollment, 31% at follow-up 1, and 31% at follow-up 2; for trend, *P* = .983).

Reinfection incidence among those reporting injecting drug use during follow-up was 2.48 per 100 PY (95% CI, 1.32–4.23; Figure 3*B*), which was higher among those reporting opioid injecting (4.73 per 100 PY; 95% CI, 1.73–10.28) as compared with stimulant injecting (2.37 per 100 PY; 95% CI, 1.19–4.25). In adjusted regression analysis, opioid-injecting drug use (aHR, 2.26; 95% CI, 1.09–6.80; *P* = .034) was associated with increased reinfection risk; no significant association with stimulant injecting was observed (Supplementary Table 5).

Reinfection incidence was 1.51 per 100 PY (95% CI, .82–2.53) among gay and bisexual men, 1.74 per 100 PY (95% CI, .83–3.19) among those reporting condomless anal intercourse with casual male partners during follow-up, and 2.37 per 100 PY (95% CI, .96, 4.90) among those reporting group sex during follow-up. In adjusted regression analyses, opioid-injecting drug use (aHR 2.09; 95% CI, 1.08–13.02; *P* = .037) was associated with increased reinfection risk; no significant association with stimulant injecting or sexual behavior among gay and bisexual men was observed (Supplementary Table 6).

### Mortality Among People With HIV-HCV Coinfection

**Figure 4.**
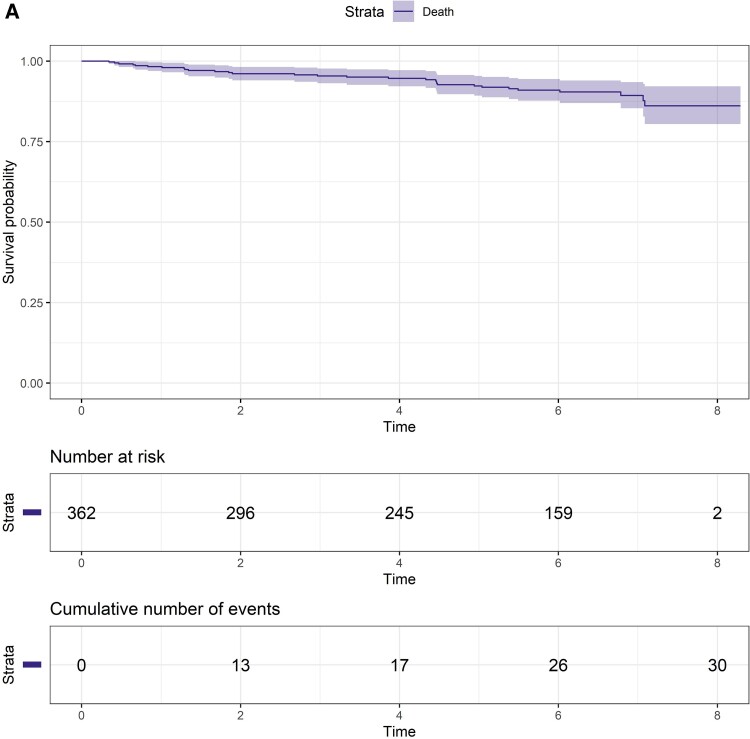

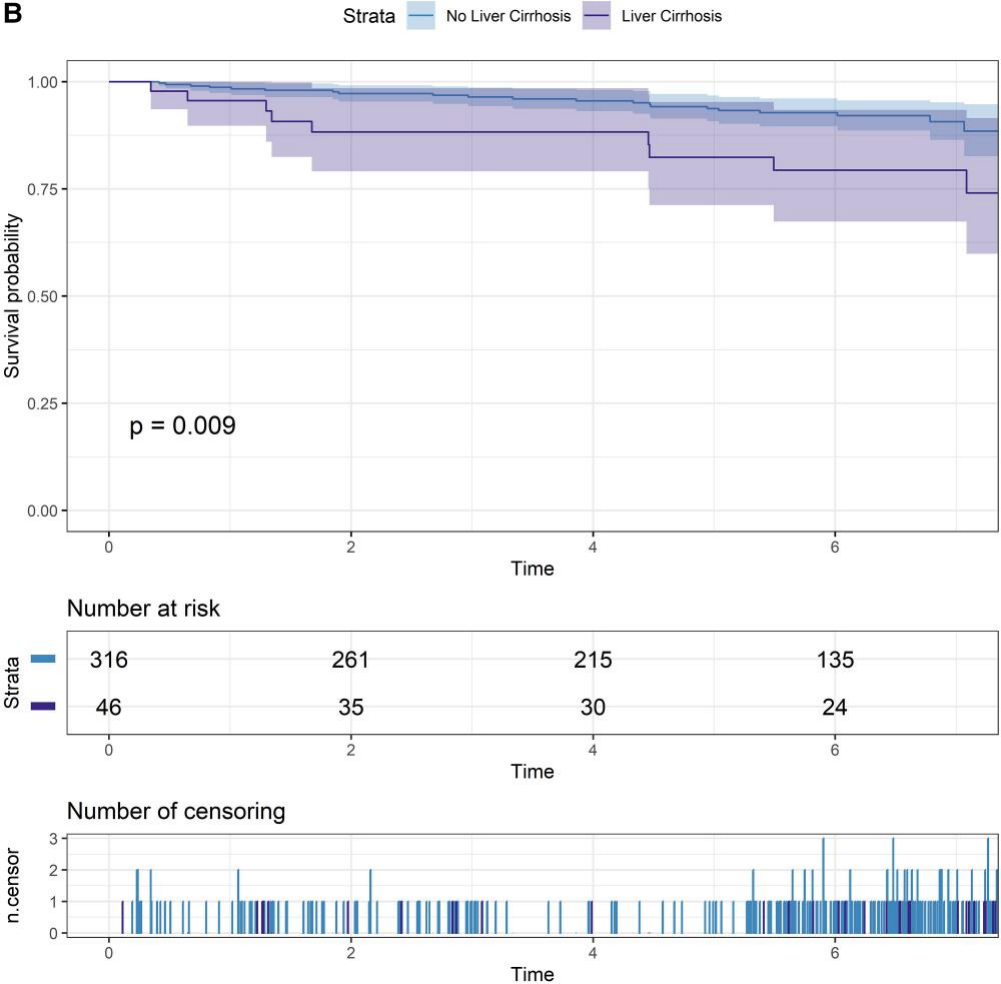

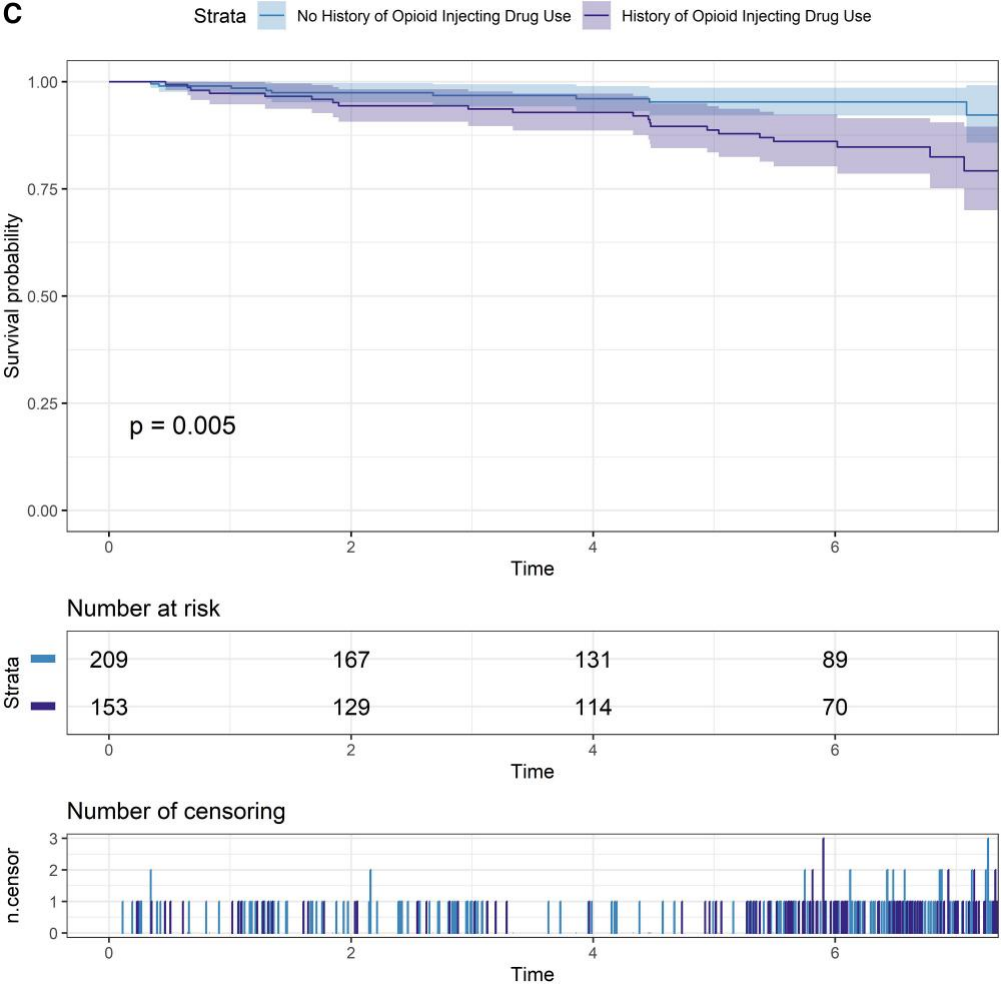
Survival probability among people with HIV–hepatitis C virus coinfection. Survival probability curves: *A*, overall; *B*, stratified by cirrhosis; *C*, opioid-injecting drug use. Shading indicates 95% CI.

Mortality incidence was 1.64 per 100 PY (95% CI, 1.11–2.34), which remained stable over time (30 deaths among 387 participants; total follow-up time, 1829 PY; median follow-up time per person, 5.67 years [IQR 2.55–6.63]; Figures 2*C* and  4*A*). Among people who died, the median age at death was 56 years (IQR, 53–57), 93% were male, the median CD4 count was 527 × 10^6^/L (IQR, 363–868), 53% injected drugs during follow-up, and 30% had cirrhosis; 17% had current HCV infection at time of death. Cancer (non-HIV and non–viral hepatitis associated, 17%) and infection (predominantly sepsis, 17%) were the most common causes of death (Supplementary Table 7). Two liver-related deaths occurred (hepatocellular carcinoma, n = 1; decompensated cirrhosis, n = 1); no liver-related deaths were reported after DAA scale-up (2016–2017). No deaths were HIV related.

Factors associated with increased mortality risk were older age (per 10-year increase; aHR, 1.67; 95% CI, 1.11–2.52), cirrhosis (aHR, 2.25; 95% CI, 1.02–4.97), and history of opioid-injecting drug use (aHR, 2.38; 95% CI, 1.04–5.43; Supplementary Table 8). Mortality incidence was 3.80 per 100 PY (95% CI, 1.74–7.22) among those with cirrhosis and 2.61 per 100 PY (95% CI, 1.39–4.47; Figure 4*B*) among those aged >60 years. Mortality incidence among those with a history of injecting drug use was 1.91 per 100 PY (95% CI, 1.26–2.77), increasing to 2.61 per 100 PY (95% CI, 1.62–3.99) among those with a history of opioid injecting (Figure 4*C*).

## DISCUSSION

Unrestricted government-subsidized access to DAAs in 2016 facilitated HCV treatment scale-up among people with HIV in Australia, with corresponding rapid and sustained declines in prevalent and incident HCV infection. With initial analysis of the CEASE cohort demonstrating a reduction in HCV RNA prevalence immediately following universal DAA access [10], additional follow-up has shown continued population-level declines. With sustained HCV viremic prevalence reduction and low reinfection incidence, CEASE has demonstrated the long-term benefits of DAA scale-up in a key population, consistent with microelimination.

CEASE has provided empirical evidence of the population-level impact of HCV treatment as prevention among people with HIV, adding to recent literature among people who inject drugs and people in prison [13–16]. Broad access to and high uptake of DAA therapy, in combination with infection prevention, are the foundations of successful HCV elimination strategies. Key features of the Australian response that enabled effective treatment scale-up and microelimination among people with HIV were a National HCV Strategy, long-standing community advocacy and engagement, high screening and diagnosis, high linkage to care and treatment, unrestricted treatment access (including retreatment of reinfection), uncapped treatment numbers per year, and no or minimal cost to the individual (A $0–$31.60/month) [8]. HCV treatment access was enhanced by the development of a diverse prescriber base (including specialists, general practitioners, and nurse practitioners in primary and tertiary care) with prescriber-focused education and training and medication dispensing through public hospital and community pharmacies. Several other high-income countries have demonstrated success with HCV elimination initiatives among people with HIV [17–21], all of which highlight the need for a strong clinical and public health policy foundation and sustained government and societal investment.

Treatment uptake and reinfection incidence reduction among the CEASE cohort were in line with the original World Health Organization targets (80% treatment uptake among those eligible, 90% reduction in incidence of new infection) [2]. HCV reinfection incidence declined as the viremic proportion fell, highlighting the individual- and population-level benefits of HCV cure (SVR), including prevention of transmission. Importantly, this was despite sustained levels of injecting drug use. Some cases of reinfection are inevitable among at-risk groups; however, successful retreatment ensured that population-level reinfection risk continued to decease, emphasizing the importance of surveillance and retention in care. While rapid DAA scale-up and low HCV reinfection incidence have been demonstrated among people with HIV (predominantly gay and bisexual men), the high prevalence of ongoing sexual and drug use risk behaviors means that reintroduction of infection remains possible [22, 23]. While treatment was scaled up rapidly among people with HIV, treatment expansion among people who inject drugs has occurred at a slower pace with high rates of reinfection reported, notably among those in prison [24]. Given the overlap of populations of people with sexual and drug use risk behaviors, continued monitoring of primary infection and reinfection incidence will be crucial in all populations and settings demonstrating microelimination success.

All-cause mortality among people in the CEASE cohort did not change significantly over time, with most being non-HCV related and all deaths being non-AIDS related. Mortality incidence in CEASE was more than double non–AIDS-related mortality in another contemporary Australian HIV cohort (AHOD; 1997–2017, 0.66 per 100 PY), although the median age was 10 years younger in AHOD (38 years) than CEASE (49 years) [25]. Notably, HCV coinfection was associated with a higher risk of death among people with HIV in the AHOD cohort [25]. Cancer (non-HIV, non–viral hepatitis related) and infection were the most common causes of death in the CEASE cohort, occurring in the context of an aging population. While only 2 liver-related deaths were reported, cirrhosis was associated with increased mortality risk. Cirrhosis may contribute to deaths attributed to bacterial infection and other chronic disease despite not being the primary cause of death, highlighting the need for ongoing advanced liver disease care for people with cirrhosis after HCV cure [26, 27]. More broadly, comprehensive management of chronic comorbidities through integrated models of care will be crucial for this population, irrespective of liver disease stage. Addressing the social determinants of health and implementing targeted interventions to manage multimorbidity will be essential steps toward reducing mortality.

While the CEASE cohort comprised an estimated 15% to 20% of the Australian HIV-HCV coinfected population, a potential limitation is the extent to which the findings are generalizable to the broader population with HIV. The cohort demographics and drug use and sexual behavior characteristics are consistent with those of the Australian HIV population, particularly gay and bisexual men [7, 28–30]. While detailed information on sexual behavior and drug use among the Australian HIV-HCV population is limited outside of this cohort, characteristics observed in CEASE were consistent with those reported in another Australian study [31]. Of note, the sexual behavior change in the CEASE cohort was reflective of wider modification among gay and bisexual men before and during the COVID-19 pandemic: there was an increase in reported higher-risk sexual behaviors among gay and bisexual men in Australia following uptake of HIV preexposure prophylaxis (government subsidized in April 2018) [29], followed by adjustments in behavior with fewer sex partners and declining HIV preexposure prophylaxis use during COVID-19 [32]. Inevitably, a proportion of the CEASE cohort was lost to follow-up (with COVID-19–related restrictions on research and clinical care particularly affecting follow-up 2). However, participant characteristics, HCV treatment uptake, and HCV RNA status at last study visit were similar among those who did and did not complete follow-up (Supplementary Tables 2–4). Additionally, CEASE enrolled people with HIV who had a past or current HCV infection to minimize bias inherent in enrolling participants interested only in HCV treatment and to ensure that all people at risk of reinfection were able to contribute. Yet, the enrolled population may have remained biased toward those more likely to initiate treatment. Furthermore, the results reflect the clinical practice of high-caseload services in urban settings—most notably in Sydney, which has the largest population of people with HIV in Australia [33]—and may be less applicable in rural or remote centers or among populations less engaged with health care. Regardless, this study suggests that HCV microelimination is feasible, and it provides important data to guide health policy supporting universal access to treatment/retreatment, decentralized HCV treatment for people with HIV, and the need for integrated care services to manage chronic comorbidities among people with HIV after HCV cure to reduce mortality.

Universal access to DAA therapy permitted rapid HCV treatment scale-up among people with HIV in Australia, with resultant sustained reductions in HCV viremic prevalence and reinfection incidence consistent with “elimination as a public health threat.” CEASE has provided further evidence in support of the population-level impact of HCV treatment as prevention and the components necessary to implement a successful microelimination initiative. The development of DAA therapy has revolutionized clinical care and generated a global impetus for elimination; ensuring access to testing, treatment, and prevention for all people with or at risk of HCV infection is essential. Striving for HCV elimination requires a broad lens focusing on health equity, universal health care, and multidisciplinary collaboration.

## Supplementary Data

Supplementary materials are available at *Open Forum Infectious Diseases* online. Consisting of data provided by the authors to benefit the reader, the posted materials are not copyedited and are the sole responsibility of the authors, so questions or comments should be addressed to the corresponding author.

## Supplementary Material

ofae665_Supplementary_Data

## Notes

***Acknowledgments.*** The authors acknowledge the contribution of the CEASE team members and CEASE protocol steering committee members who are not coauthors of this article. The authors also acknowledge the clinical services participating in CEASE. Data for CEASE are gathered from 12 primary care services and 2 tertiary health services. The CEASE study was supported by Gilead Sciences, Inc and the Australian Government Department of Health and Ageing.

The authors thank the study participants for their contribution to the research, as well as current and past researchers and staff. The authors acknowledge members of the study group.

*Protocol Steering Committee* **—**Gail Matthews (chair, Kirby Institute, University of New South Wales [UNSW] Sydney, Sydney, Australia), David Baker (East Sydney Doctors, Sydney, Australia), Mark Bloch (Holdsworth House Medical Practice, Sydney, Australia), Joanne Carson (Kirby Institute, UNSW Sydney, Sydney, Australia), Gregory Dore (Kirby Institute, UNSW Sydney, Sydney, Australia), Joseph Doyle (Burnet Institute, Melbourne, Australia), Tim Duck (NSW Health, Sydney, Australia), Robert Finlayson (Taylor Square Private Clinic, Sydney, Australia), Margaret Hellard (Burnet Institute, Melbourne, Australia), Vanessa Towell (Australasian Society for HIV, Viral Hepatitis and Sexual Health Medicine, Sydney, Australia), Sarah Lambert (AIDS Council of NSW, Sydney, Australia), Stuart Loveday (Hepatitis NSW, Sydney, Australia), Pip Marks (Kirby Institute, UNSW Sydney, Sydney, Australia), Marianne Martinello (Kirby Institute, UNSW Sydney, Sydney, Australia), Jeffrey Post (Albion Centre, Sydney, Australia), Leila Stennett (Australian Federation of AIDS Organisations, Sydney, Australia), Joseph Sasadeusz (Royal Melbourne Hospital, Melbourne, Australia).

*Coordinating Centre, Kirby Institute, UNSW, Sydney, Australia* **—**Gail Matthews (coordinating principal investigator), Gregory Dore (coordinating principal investigator), Ecaterina Filep (data manager), Joanne Carson (study coordinator), Pip Marks (clinical trials manager), Marianne Martinello (senior research fellow, statistician), Kathy Petoumenos (statistician), Ineke Shaw (systems manager), Jasmine Yee (study coordinator), Lanni Lin (study coordinator).

*Site principal investigators* **—**New South Wales: Eva Jackson (Blue Mountains Sexual Health, Sydney, Australia), Nicholas Doong (Dr Doong's Surgery, Sydney, Australia), David Baker (East Sydney Doctors, Sydney, Australia), Mark Bloch (Holdsworth House Medical Practice, Sydney, Australia), Phillip Read (Kirketon Road Centre, Sydney, Australia), Archana Sud (Nepean Sexual Health, Sydney, Australia), Gregory Dore (St Vincent's Hospital, Sydney, Australia), Anna McNulty (Sydney Sexual Health Centre, Sydney, Australia), Robert Finlayson (Taylor Square Private Clinic, Sydney, Australia), Jeffrey Post (Albion Centre, Sydney, Australia), Shailendra Sawleshwarkar (Western Sydney Sexual Health, Sydney, Australia). Queensland: Diane Rowling and Leo Van Lieshout (Brisbane Sexual Health Clinic, Brisbane, Australia). South Australia: David Shaw (Royal Adelaide Hospital, Adelaide, Australia). Victoria: Richard Moore (Northside Clinic, Melbourne, Australia).

*Site coordinators* **—**New South Wales: Vincenzo Fragomeli and Joanne Gough (Blue Mountains Sexual Health, Sydney, Australia); Shane Hewitt (Dr Doong's Surgery, Sydney, Australia); Roshanak Mousavi and Melissa Benson (East Sydney Doctors, Sydney, Australia); Annabelle Caspersz and Jackson Thorne (Holdsworth House Medical Practice, Sydney, Australia); Rosie Gilliver and Ed Silins (Kirketon Road Centre, Sydney, Australia); Vincenzo Fragomeli (Nepean Sexual Health, Sydney, Australia); Alice Hawkes, Alison Sevehon, Fiona Peet, and Rebecca Hickey (St Vincent's Hospital, Sydney, Australia); Ruthy McIver and Anik Ray (Sydney Sexual Health Centre, Sydney, Australia); Piper Calleia, Jaqueline Le, Lynn Tan, and Ching Tan (Taylor Square Private Clinic, Sydney, Australia); Diane Smith and Raghib Ahmad (Albion Centre, Sydney, Australia); Yvette Ormsby, Holly Miller, and Tichaona Jaricha (Western Sydney Sexual Health, Sydney, Australia). Queensland: Fiona Taylor and Sonia Benn (Brisbane Sexual Health Clinic, Brisbane, Australia). South Australia: Jane Rose and Catherine Ferguson (Royal Adelaide Hospital, Adelaide, Australia). Victoria: Susan Boyd and Sian Gowalds (Northside Clinic, Melbourne, Australia).

***Author contributions.*** M. M., J. J. P., R. F., D. B., P. R., D. S., M. B., J. D., M. H., G. J. D., and G. V. M. contributed to study design, conceived the study proposal, and developed the methodology. M. M., J. J. P., R. F., D. B., P. R., D. S., M. B., G. J. D., and G. V. M. were involved in participant recruitment and data collection. E. F., S. H.-H., and J. M. C. contributed to study administration and data curation. J. M. C. conducted the data analysis, with oversight from M. M. M. M. and J. M. C. led the writing of the manuscript, with critical revision by G. J. D. and G. V. M. All authors have seen and approved the final version of the manuscript.

***Financial support*.** The Kirby Institute is funded by the Department of Health and Ageing, Australian Government. Research reported in this publication was supported by Gilead Sciences as an investigator-initiated study.
