# Supplementary material for: Control and Elimination of Hepatitis C Virus Among People With HIV in Australia: Extended Follow-up of the CEASE Cohort (2014–2023)
Source: Open Forum Infect Dis. 2024 Dec 17;11(12):ofae665. doi: 10.1093/ofid/ofae665 (PMC11650530; doi:10.1093/ofid/ofae665)
Supplement: ofae665_Supplementary_Data [file ofae665_supplementary_data.docx]

**Supplementary Material**

**Control and elimination of HCV among people with HIV in Australia: the CEASE study (2014-2023)**

Marianne Martinello*^1,2^, Joanne Carson*^1^, Jeffrey Post^2,3,4^, Robert Finlayson^5^, David Baker^6^, Phillip Read^1,7^, David Shaw^8^, Mark Bloch^9^, Joseph Doyle^10,11^, Margaret Hellard^10,11^, Ecaterina Filep^1^, Samira Hosseini-Hooshyar^1^, Gregory Dore^1,12^, Gail Matthews^1,12^ on behalf of the CEASE study group

*Joint first author

^1^The Kirby Institute, University of New South Wales, Sydney, Australia; ^2^Prince of Wales Hospital, Sydney, Australia; ^3^The Albion Centre, Sydney, Australia; ^4^School of Clinical Medicine, University of New South Wales, Sydney, Australia; ^5^Taylor Square Private Clinic, Sydney, Australia; ^6^East Sydney Doctors, Sydney, Australia; ^7^Kirketon Road Clinic, Sydney, Australia; ^8^Royal Adelaide Hospital, Adelaide, Australia; ^9^Holdsworth House Medical Practice, Sydney, Australia; ^10^Alfred Hospital, Melbourne, Australia; ^11^Burnet Institute, Melbourne, Australia; ^12^St Vincent's Hospital, Sydney, Australia.

**Methods**

*Study assessments*

The enrolment questionnaire included demographics (age, gender, sexual identity, ethnicity, education, main source of income and accommodation), drug and alcohol use, sexual behaviour (completed by gay and bisexual men only) and HCV acquisition, knowledge and treatment willingness. Non-injecting drug use and injecting drug use history were collected for lifetime (ever), previous six months (current) and the previous month (recent). Recent (previous month) associated risk behaviours including use of a new sterile needle/syringe for all injections, needle/syringe borrowing and lending, and ancillary injecting equipment sharing were also collected. Information regarding sexual behaviour was collected for the previous six months, and included presence of regular and/or casual male partners, total number of male partners, sexual practices (including group sex and condom-less anal intercourse) and HIV and HCV disclosure with male partners. HCV specific questions, including mode of acquisition, year of diagnosis and treatment history, comprised the final component. HCV treatment willingness and HCV treatment intent were assessed using a five-point Likert scale.

HCV treatment (both prospective and retrospective), including regimen, date of initiation and cessation, duration and outcome, were recorded by site study staff. Local HCV RNA results (quantitative or qualitative, assay type, genotype [if available]) were collected prior to and following treatment.

*Study* *definitions*

Injecting drug use was defined as “never” (no history of injecting drug use), “ever” (history of injecting drug use, with no use in the six months prior to the study visit), “current” (injecting drug use within six months of the study visit) and “recent” (injecting drug use within one month of the study visit).

Sexual risk behaviour among gay and bisexual men was defined as “no or low risk” (no regular or casual male partners; HIV-negative, HCV-negative regular male partner only [with or without anal intercourse]; HIV-positive or HCV-positive regular male partner only, condom use for all anal intercourse), “intermediate risk” (condom-less anal intercourse with HIV-positive/unknown or HCV-positive/unknown regular male partner; one or more casual male partner/s with condom use for all anal intercourse) and “highest risk” (condom-less anal intercourse with one or more casual male partners, including group sex).

Liver fibrosis stage was assessed by liver biopsy or transient elastography (Fibroscan®). For transient elastography, the following cut-off values were used: F0/F1, <7.1 kPa; F2, 7.1 – 9.4 kPa; F3, 9.5 – 12.4 kPa; F4, ≥12.5 kPa (1, 2). Cirrhosis was defined as Fibroscan® >12.5 kPa or liver biopsy equal to Metavir F4.

*Laboratory* *methods*

HCV RNA status was determined locally (standard-of-care) and centrally (via dried blood spot). HCV RNA was extracted from DBS using the NucliSens^®^ easyMAG™ system (BioMérieux, France). Reverse transcription of RNA with random hexamers was performed using the Invitrogen Superscript™ system (Vilo IV), and Core-E2 (3) and NS5A (4) regions were amplified by polymerase chain reaction. Sanger sequencing was performed at the Australian Genome Research Facility on the Applied Biosystems™ 3730xl DNA Analyzer. Sequence curation was performed using RECall (5). DBS is not offered as a clinically approved test in Australia or included as standard of care and was collected for research purposes. If discrepancies were noted between standard-of care and central laboratory samples noted, study sites were asked to confirm local results, provide additional data or collect venous samples for centralised retesting.

*Statistical analysis*

Causes of death were categorised using HIV Cohorts Data Exchange Protocol (HICDEP) codes (6): HIV-related (AIDS-defining infections and malignancies; HICDEP category 01 and subcategories); infection (infections other than AIDS-defining infections; category 02); cancer (non-HIV or viral hepatitis associated malignancies [excluding hepatocellular carcinoma]; category 04); liver (chronic viral hepatitis, hepatocellular carcinoma, and liver failure; categories 03, 04.20, and 14); cardiovascular (acute myocardial infarction or other ischaemic heart disease, stroke, and other heart or vascular disease; categories 08, 09, and 24); respiratory (chronic obstructive lung disease and other respiratory diseases; categories 13 and 25); substance use (active use, including overdose; category 19); violent death (suicide, accident, or other violent death; categories 16 and 17); haematological disease (category 20); unknown or unclassifiable (categories 91 and 92); and other (category 90).

**Reference List**

1. Ziol M, Handra-Luca A, Kettaneh A, Christidis C, Mal F, Kazemi F, et al. Noninvasive assessment of liver fibrosis by measurement of stiffness in patients with chronic hepatitis C. Hepatology. 2005;41(1):48-54.

2. de Ledinghen V, Douvin C, Kettaneh A, Ziol M, Roulot D, Marcellin P, et al. Diagnosis of hepatic fibrosis and cirrhosis by transient elastography in HIV/hepatitis C virus-coinfected patients. Journal of acquired immune deficiency syndromes (1999). 2006;41(2):175-9.

3. Lamoury FM, Jacka B, Bartlett S, Bull RA, Wong A, Amin J, et al. The Influence of Hepatitis C Virus Genetic Region on Phylogenetic Clustering Analysis. PloS one. 2015;10(7):e0131437.

4. Lindstrom I, Kjellin M, Palanisamy N, Bondeson K, Wesslen L, Lannergard A, et al. Prevalence of polymorphisms with significant resistance to NS5A inhibitors in treatment-naive patients with hepatitis C virus genotypes 1a and 3a in Sweden. Infectious diseases (London, England). 2015;47(8):555-62.

5. Woods CK, Brumme CJ, Liu TF, Chui CKS, Chu AL, Wynhoven B, et al. Automating HIV Drug Resistance Genotyping with RECall, a Freely Accessible Sequence Analysis Tool. Journal of clinical microbiology. 2012;50(6):1936-42.

6. EuroCoord. HIV Cohorts Data Exchange Protocol [Available from: <https://hicdep.org/Wiki/About-HICDEP>.

**Supplementary Table 1.** Details of sites participating in CEASE

| **Clinic** | **Location** | **Type of service/s** |
| --- | --- | --- |
| Blue Mountains Sexual Health | Sydney, NSW | Sexual health clinic |
| Brisbane Sexual Health Clinic | Brisbane, Qld | Sexual health clinic |
| Dr Doong’s Surgery | Sydney, NSW | General practice |
| East Sydney Doctors | Sydney, NSW | General practice |
| Holdsworth House | Sydney, NSW | General practice |
| Kirketon Road Centre | Sydney, NSW | Community health clinic |
| Nepean Sexual Health | Sydney, NSW | Sexual health clinic |
| Northside Clinic | Melbourne, Vic | General practice |
| Royal Adelaide Hospital | Adelaide, SA | Specialist viral hepatitis service |
| St Vincent’s Hospital | Sydney, NSW | Specialist viral hepatitis service |
| Sydney Sexual Health Centre | Sydney, NSW | Sexual health clinic |
| Taylor Square Private Clinic | Sydney, NSW | General practice, sexual health clinic |
| The Albion Centre | Sydney, NSW | Sexual health clinic |
| Western Sydney Sexual Health | Sydney, NSW | Sexual health clinic |

**Supplementary Table 2.** Study termination

| **Reason for study termination** | **N** | **HCV RNA positive at enrolment n (%)** | **HCV RNA positive at last follow-up n (%)** | **HCV treatment by last follow up^#^ n (%)** |
| --- | --- | --- | --- | --- |
| Completed study^ | 253 | 217 (86) | 1 (0.5) | 212 (98) |
| Death* | 24 | 17 (71) | 5 (21) | 14 (82) |
| Lost to follow up after enrolment | 40 | 30 (75) | 22 (55) | 10 (33) |
| Lost to follow up after follow up 1 | 85 | 77 (91) | 3 (4) | 76 (99) |
| **Total** | 402 | 341 (85) | 31 (8) | 312 (92) |

*Total deaths in CEASE cohort, n=30. Six participants died after completing follow-up 2.

^^^32 participants had follow-up 2 impacted by restrictions imposed by COVID; available clinical data was more limited (including lack of HCV RNA)

^#^Among people with current HCV infection (HCV RNA positive) at enrolment

**Supplementary Table 3.** Characteristics of participants who attended CEASE follow-up visits

|  | **Enrolment** | **Follow Up 1** | **Follow Up 2** | ***P*** |
| --- | --- | --- | --- | --- |
|  | N=402 | N=347 | N=268 |  |
| Age at enrolment, median (IQR) | 49 (43, 55) | 49 (42, 55) | 50 (43, 55) | 0.813 |
| Male, n (%) | 382 (95) | 329 (95) | 254 (95) | 0.987 |
| Gay and bisexual men, n (%) | 335 (83) | 287 (83) | 223 (83) | 0.973 |
| White, n (%) | 344 (86) | 302 (87) | 242 (90) | 0.194 |
| Full or part-time employment, n (%) | 154 (38) | 124 (36) | 91 (34) | 0.500 |
| Completed higher education, n (%)^1^ | 207 (52) | 171 (49) | 130 (48) | 0.716 |
| Lives alone, n (%) | 176 (44) | 150 (43) | 117 (44) | 0.971 |
| Years since HIV diagnosis, median (IQR) | 15 (8, 22) | 15 (8, 23) | 15 (9, 23) | 0.538 |
| CD4 count at enrolment, median (IQR) | 596  (440, 810) | 592  (422, 794) | 610  (435, 811) | 0.842 |
| HCV RNA detected at enrolment, n (%) | 341 (85) | 303 (87) | 229 (85) | 0.607 |
| LSM at enrolment, median (IQR) | 6.3 (5.0, 8.4) | 6.3 (5.0, 8.4) | 6.1 (4.9, 8.1) | 0.933 |
| Cirrhosis, n (%) | 51 (13) | 46 (13) | 33 (12) | 0.607 |
| IDU ever, n (%) | 331 (82) | 292 (84) | 224 (84) | 0.723 |
| IDU within 6 months of enrolment, n (%) | 146 (36) | 128 (37) | 98 (37) | 0.987 |
| CAI CMP, n (%)* | 173 (52) | 143 (50) | 109 (48) | 0.800 |
| Group sex, n (%)* | 108 (32) | 87 (30) | 70 (31) | 0.837 |

*Among gay and bisexual men

Abbreviations: CAI CMP, condomless anal intercourse with casual male partners; IDU, injecting drug use; LSM, liver stiffness measurement

**Supplementary Table 4.** Characteristics of participants who were and were not retained in follow up

|  | **Retained in follow up** | **Lost to follow up** | ***P*** |
| --- | --- | --- | --- |
|  | N=277 | N=125 |  |
| Age at enrolment, median (IQR) | 50 [44, 55] | 48 [41, 54] | 0.101 |
| Male, n (%) | 262 (95) | 120 (96) | 0.722 |
| Gay and bisexual men, n (%) | 230 (83) | 105 (84) | 0.923 |
| White, n (%) | 247 (89) | 97 (77) | 0.004 |
| Born in Australia, n (%) | 188 (68) | 70 (56) | 0.020 |
| Full or part-time employment, n (%) | 93 (33) | 61 (49) | 0.005 |
| Completed higher education, n (%) ^1^ | 134 (48) | 73 (58) | 0.079 |
| Treated in primary care, n (%) | 170 (61) | 66 (53) | 0.132 |
| HCV RNA detected at enrolment, n (%) | 234 (85) | 107 (86) | 0.888 |
| Received HCV treatment ever, n (%) | 226 (82) | 86 (69) | 0.007 |
| Cirrhosis, n (%) | 37 (13) | 14 (11) | 0.660 |
| IDU ever, n (%) | 235 (85) | 96 (77) | 0.070 |
| IDU within 6 months of enrolment, n (%) | 104 (38) | 42 (34) | 0.516 |
| CAI CMP, n (%)* | 113 (49) | 60 (57) | 0.214 |
| Group sex, n (%)* | 73 (32) | 36 (34) | 0.737 |

*Among gay and bisexual men

Characteristics of participants attending each follow visit were similar (**Supplementary Table 3**). When comparing the population retained in follow up vs. those lost to follow up **(Supplementary Table 4)**, those lost to follow up comprised a lower proportion of white individuals, lower proportion of Australian born individuals and higher proportion of people who were employed on a full or part time basis. However, it is unclear whether this is reflective of decreased engagement with care among overseas born or migrant populations or whether it is reflective temporary residence status (travel, work or study) and population mobility (both within Australia and internationally).

**Supplementary Table 5**. Factors associated with reinfection among people with HIV

| **Variable** | **HR (95% CI)** | ***P*** | **aHR (95% CI)** | ***P*** |
| --- | --- | --- | --- | --- |
|  |  |  |  |  |
| **Gay or Bisexual Man** |  |  |  |  |
| No | 1 | - | - | - |
| Yes | 1.42 (0.32, 6.26) | 0.466 | - | - |
| **HIV RNA per mm³** |  |  |  |  |
| ³ 50 copies | 1 | - | 1 | - |
| < 50 copies | 4.51 (1.64,12.40) | 0.004 | 1.99 (0.56, 7.06) | 0.558 |
| **CD4 count per mm³** |  |  |  |  |
| ³ 200 cells | 1 | - | - | - |
| < 200 cells | 1.06 (0.37, 3.05) | 0.923 | - | - |
| **Stimulant injecting during follow-up** |  |  |  |  |
| No | 1 | - | 1 | - |
| Yes | 3.04 (1.06, 8.75) | 0.039 | 2.26 (0.75, 6.80) | 0.148 |
| **Opioid injecting during follow-up** |  |  |  |  |
| No | 1 | - | 1 | - |
| Yes | 2.97 (1.36, 6.49) | 0.006 | 3.17 (1.09, 9.23) | 0.034 |

**Supplementary Table 6**. Factors associated with reinfection among gay and bisexual men with HIV

| **Variable** | **HR (95% CI)** | ***P*** | **aHR (95% CI)** | ***P*** |
| --- | --- | --- | --- | --- |
|  |  |  |  |  |
| **Age** |  |  |  |  |
| Per 10 years | 0.79 (0.46, 1.35) | 0.391 | - | - |
| **HIV RNA per mm³** |  |  |  |  |
| ³ 50 copies | 1 | - | - | - |
| < 50 copies | 2.17 (0.49, 9.71) | 0.309 | - | - |
| **CD4 count per mm³** |  |  |  |  |
| ³ 200 cells | 1 | - | - | - |
| < 200 cells | NA | NA | - | - |
| **Stimulant injecting during follow-up** |  |  |  |  |
| No | 1 | - | 1 | - |
| Yes | 3.04 (1.06, 8.75) | 0.031 | 2.75 (0.72, 10.60) | 0.140 |
| **Opioid injecting during follow-up** |  |  |  |  |
| No | 1 | - | 1 | - |
| Yes | 3.94 (1.24, 12.60) | 0.021 | 2.09 (1.08, 13.02) | 0.037 |
| **CAI-CMP during follow-up** |  |  |  |  |
| No | 1 | - | - | - |
| Yes | 1.60 (0.50, 5.10) | 0.427 | - | - |
| **Group-sex during follow-up** |  |  |  |  |
| No | 1 | - | - | - |
| Yes | 2.20 (0.77, 6.28) | 0.140 | 2.18 (0.70, 6.77) | 0.178 |

Abbreviation: CAI CMP, condomless anal intercourse with casual male partners

**Supplementary Table 7**. Causes of death

| **Cause of death** | **N** |
| --- | --- |
| HIV-related | 0 |
| Infection | 5 (17) |
| Cancer (non-HIV, non-viral hepatitis associated) | 5 (17) |
| Liver | 2 (7) |
| Respiratory | 2 (7) |
| Substance use | 2 (7) |
| Violent death | 2 (7) |
| Cardiovascular | 1 (3) |
| Haematological | 1 (3) |
| Unknown or unclassifiable | 10 (33) |

Cause of death was assigned by the site investigator and categorised based on HICDEP codes (6).

Six participants died after completing follow-up 2 visit.

**Supplementary Table 8**. Factors associated with mortality among people with HIV-HCV co-infection

| **Variables** | **HR (95% CI)** | ***P*** | **aHR (95% CI)** | ***P*** |
| --- | --- | --- | --- | --- |
|  |  |  |  |  |
| **Age** |  |  |  |  |
| Per 10 years | 1.71 (1.16, 2.53) | 0.007 | 1.67 (1.11, 2.52) | 0.013 |
| **Cirrhosis** |  |  |  |  |
| No | 1 | - | 1 | - |
| Yes | 2.88 (1.32, 6.29) | 0.007 | 2.25 (1.02, 4.97) | 0.045 |
| **HIV RNA per mm³** |  |  |  |  |
| ³ 50 copies | 1 | - | - | - |
| < 50 copies | 1.22 (0.47, 3.19) | 0.688 | - | - |
| **CD4 count per mm³** |  |  |  |  |
| ³ 200 cells | 1 | - | - | - |
| < 200 cells | 0.91 (0.12, 6.65) | 0.923 | - | - |
| **Tobacco smoking** |  |  |  |  |
| Never | 1 | - | 1 | - |
| Ever | 2.67 (0.93, 7.65) | 0.068 | 1.69 (0.56, 5.11) | 0.352 |
| **Opioid injecting** |  |  |  |  |
| Never | 1 | - | 1 | - |
| Ever | 2.97 (1.36, 6.49) | 0.006 | 2.38 (1.04, 5.43) | 0.040 |

**Supplementary Figure 1**. HCV treatment initiation among people with HIV-HCV co-infection between 2000 - 2023, by regimen type.

Abbreviation: DAA, direct-acting antiviral

**Supplementary Figure 2. Longitudinal drug use and sexual behaviour among CEASE participants**

Proportion of participants reporting (A) injecting and non-injecting drug use, and gay and bisexual men reporting (B) sexual risk behaviour.

Abbreviations: 1m, one month; 6m, six months; CAI, condomless anal intercourse; IDU, injecting drug use.
